# Prevalence, Geographic Variations, and Determinants of Pain Among Older Adults in China: Findings from the National Urban and Rural Elderly Population (UREP) Survey

**DOI:** 10.3390/healthcare13212720

**Published:** 2025-10-28

**Authors:** Ge Yan, Yutong Wu, Hui Zhang, Zhimeng Jia, Xiaohong Ning, Chen Wang

**Affiliations:** 1 School of Population Medicine and Public Health, Chinese Academy of Medical Sciences, Peking Union Medical College, Beijing 100006, China; b2022027016@pumc.edu.cn; 2 Institute of Acoustics, University of Chinese Academy of Social Sciences, Beijing 102488, China; 3 Department of Nursing, Peking Union Medical College Hospital, Chinese Academy of Medical Sciences, Beijing 100730, China; 4 Temmy Latner Centre for Palliative Care, Toronto, ON M5T 3L9, Canada; 5 Department of Family and Community Medicine, University of Toronto, Toronto, ON M5S 1A1, Canada; 6 Program in Global Palliative Care, Department of Global Health and Social Medicine, Harvard Medical School, Boston, MA 02215, USA; 7 Palliative Medicine Center, Peking Union Medical College Hospital, Chinese Academy of Medical Sciences, Peking Union Medical College, Beijing 100730, China

**Keywords:** pain prevalence, pain management, multimorbidity pattern, healthy aging

## Abstract

**Objectives:** This study aimed to reveal the prevalence, geographic variations, and determinants of pain among the Chinese older adult population and provide empirical strategies for pain management in older adults in China. **Methods:** A total of 21,346 Chinese residents aged ≥ 60 years from 31 provinces in mainland China participated in our survey. Standardized questionnaires were used to collect data on socioeconomic characteristics, lifestyle factors, and self-reported pain experiences. Multivariate logistic regression models were used to estimate the associations between individual socioeconomic status, chronic diseases, and pain. **Results:** The national prevalence of pain was 56.5% (95% *CI*: 55.9–57.1%), representing approximately 140 million Chinese older adults. The prevalence increased with aging and peaked at 80 years and older (61.00%, 95% *CI*: 59.30–62.70%). Women (62.36%, 95% *CI*: 61.47–63.25%), rural residents (61.27%, 95% *CI*: 60.34–62.20%), and those with no formal education (65.08%, 63.90–66.26%) had a higher prevalence than men (50.27%, 95% *CI*: 49.32–51.22%), urban residents (52.19%, 95% *CI*: 51.28–53.10%), and those with higher education levels, respectively. Provincial prevalence ranged from 38.98% in Shanghai to 72.75% in Gansu Province. The presence of chronic diseases significantly increased the odds of pain, with multimorbidity (three or more chronic diseases) showing the strongest association (*OR* = 11.380, 95% *CI*: 10.257–12.627). **Conclusions and Implications:** Pain was highly prevalent among older adults in China and varied geographically. Socioeconomic status, chronic diseases, and multimorbidity were strongly associated with pain prevalence. Our findings support prioritizing the reduction in gender and geographic disparities in China’s pain management strategies. An integrated approach addressing both pain and chronic diseases should be urgently established in China’s healthcare system for older adults.

## 1. Introduction

Pain, defined as an unpleasant sensory and emotional experience associated with actual or potential tissue damage [1,2], represents a significant global health challenge. It is associated with reduced quality of life, increased healthcare utilization, and a substantial economic burden [3,4,5,6]. The World Health Organization emphasizes addressing pain management in the elderly as a critical component of healthy aging strategies [3,7]. From a life course perspective, health risks accumulate from the embryonic period and intensify throughout the aging process, leading to complex multisystem health problems [8], with chronic pain emerging as a prominent manifestation of this cumulative burden in older adults. As one of the world’s most populous nations with a rapidly aging population, understanding the landscape of pain among older adults in China is crucial for public health planning and policymaking.

Previous studies have reported varying prevalence rates of pain in older adults, ranging from 36.6% to 76% across different countries [9,10,11,12,13]. In the United States, a national health survey found that 52.9% of adults aged 65 and older reported pain in the past month [14]. A large-scale study in the United Kingdom reported a chronic pain prevalence of 66.2% among adults aged 50 and over [15]. In Japan, a population-based study revealed that 39.3% of adults aged 20 and older experienced chronic pain [16]. In China, research on pain prevalence among older adults has been limited. A cross-sectional study China reported a chronic body pain prevalence of 35.9% among general adults [17]. Another study found a prevalence of 80.08% for chronic pain in Chinese disabled elderly people [18]. These variations likely reflect differences in study methodologies, pain definitions, and population characteristics. However, nationally representative data on pain prevalence, its geographic distribution, and associated factors among older adults in China remain limited.

China’s vast territory and diverse socioeconomic landscape present unique challenges and opportunities for studying pain in older adults. The country’s rapid urbanization, regional economic disparities, and varying healthcare resources may contribute to differences in pain prevalence and management across regions [19,20]. Understanding these geographic variations is crucial for developing targeted interventions and allocating resources effectively.

Despite the growing body of research on pain in older adults globally, comprehensive nationwide studies in China remain scarce. This study addresses this gap using data from the national Urban and Rural Elderly Population (UREP) survey to investigate the prevalence, geographic variations, and determinants of pain among older adults in China. The aim of this study was to (1) determine the national prevalence of pain among Chinese adults aged 60 years and older using data from the fourth national Urban and Rural Elderly Population (UREP) survey; (2) examine geographic variations in pain prevalence across 31 provinces; (3) identify sociodemographic, lifestyle, and health-related factors associated with pain; and (4) investigate the relationship between multimorbidity patterns and pain severity. These objectives will provide essential evidence for developing targeted pain management strategies and health policies for China’s aging population.

## 2. Methods

### 2.1. Study Design and Participants

A cross-sectional analysis was conducted using data from the fourth national Urban and Rural Elderly Population (UREP) survey. Initiated by the China National Committee on Aging (CNCA) in 2000, the UREP survey is a quinquennially administered, nationally representative survey of the Chinese population aged 60 years and older [21].

Inclusion criteria were (1) age ≥ 60 years at the time of survey; (2) permanent residence in the sampled community for at least 6 months; (3) ability to provide informed consent either directly or through a legal proxy; and (4) completion of the core questionnaire items including pain assessment.

Exclusion criteria were (1) severe cognitive impairment that prevented meaningful participation (as assessed by interviewers using standardized cognitive screening questions); (2) hospitalization or institutionalization at the time of survey; (3) incomplete pain assessment data.

The study protocol was approved by the Ethics Committee of the Chinese National Bureau of Statistics, and oral informed consent was obtained from all participants before interviews.

### 2.2. Sampling and Data Collection

**Figure 1:**
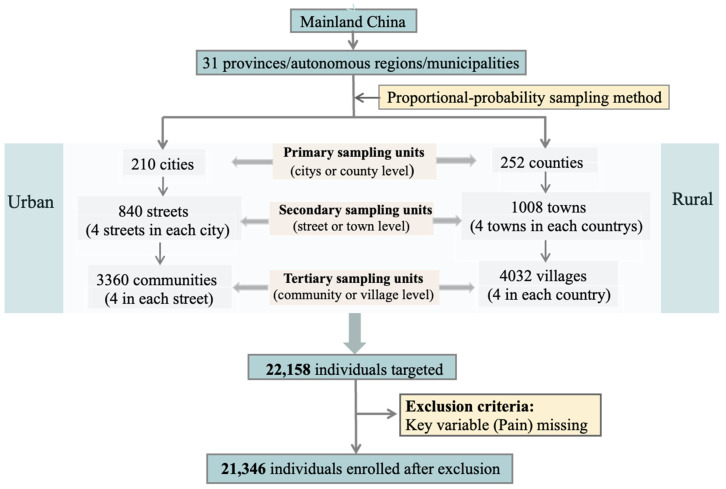
The national UREP study profile.

A multistage, stratified cluster sampling procedure was used to ensure national representativeness. Mainland China was stratified into 31 Provinces, and a three-stage cluster sampling procedure was conducted based on established administrative divisions. First, cities or counties were randomly selected as primary sampling units (PSUs). Second, streets or towns were randomly selected as secondary sampling units (SSUs) within each PSU. Third, communities or villages were randomly selected as TSUs within each SSU. Probability proportional to size (PPS) sampling was used to account for varying sizes of sampling units at each stage. Individuals aged 60 years or older were then randomly sampled from each TSU (Figure 1) [21]. Data were collected through face-to-face interviews conducted by trained interviewers using standardized questionnaires. The questionnaires collected information on sociodemographic characteristics, lifestyle factors, health status, and self-reported physician-diagnosed chronic diseases. The final study sample consisted of 21,346 participants.

### 2.3. Measures

Pain was assessed using a structured questionnaire approach adapted from validated epidemiological surveys. Participants were asked: “During the past 12 months, have you experienced any physical pain?” (yes/no). This 12-month recall period was chosen to capture both chronic and recurrent pain conditions while minimizing recall bias compared to lifetime assessments. For participants reporting pain, severity was assessed using a three-category verbal rating scale (mild, moderate, severe).

Sociodemographic variables included age (60–64, 65–69, 70–74, 75–79, 80+ years), gender (male, female), ethnicity (Han, minority), education level (illiterate, primary school, junior high school and above), marital status (married, unmarried), residence (urban, rural), and annual household income (quartiles). Lifestyle factors included smoking status (never, former, current), alcohol consumption (none or occasionally, ≥1 time/week), and physical exercise frequency (never, <1 time/week, 1–2 times/week, 3–5 times/week, ≥6 times/week). Health status was evaluated based on the presence of chronic diseases. Ten common chronic diseases were investigated: hypertension, diabetes, cardiovascular and cerebrovascular diseases, gastric disorders, osteoarthritis, chronic lung diseases, asthma, cancer, reproductive system diseases, and cataracts/glaucoma.

### 2.4. Statistical Analysis

Descriptive statistics were used to characterize the study participants. The overall and subgroup-specific prevalence of pain and their respective 95% confidence intervals (*CI*s) were calculated.

Chi-square tests were performed to examine bivariate associations between pain prevalence and each categorical variable. The Cochran–Armitage test for trend was used for ordinal variables (age groups, education level, income quintiles, exercise frequency). All percentages were calculated based on non-missing values, with missing data reported separately. Statistical significance was set at *p* < 0.05.

Multivariable logistic regression models were used to investigate the associations of sociodemographic factors, lifestyle factors, and chronic diseases with pain, adjusting for potential confounders. Odds ratios (*OR*s) and 95% *CI*s were reported. The impact of different chronic disease multimorbidity patterns on the risk of severe pain was assessed using a multinomial logistic regression model. Chronic disease patterns were categorized as no chronic disease (reference), single chronic disease, 2 chronic diseases, and 3 or more chronic diseases. *OR*s and 95% *CI*s were calculated, adjusting for other covariates. To examine the association between pain prevalence and socioeconomic development at the provincial level, a 3-level generalized linear mixed model (GLMM) was constructed. Individual-level variables were treated as fixed effects, while city/county and province were considered random effects. The GLMM analysis adjusted for individual-level covariates. All statistical analyses were conducted using R software (version 4.0.3), with a 2-tailed *p* value ≤ 0.05 considered statistically significant.

## 3. Results

### 3.1. Characteristics of the Study Population

The study included 21,346 participants aged 60 years and older from 31 Provinces in mainland China. As shown in Table 1, 34.1% were aged 60–64 years, with decreasing proportions in older age groups. Females comprised 51.7% of the sample, and 47.7% resided in rural areas. Educational attainment was generally low, with 28.4% reporting no formal education and 41.5% having completed only primary school. Most participants (78.7%) did not have basic medical insurance for urban employees.

### 3.2. Prevalence and Distribution of Pain

The overall prevalence of pain among participants was 56.5% (95% *CI*: 55.9–57.1%). Of those reporting pain, 21.4% described it as mild, 48.7% as moderate, and 28.5% as severe, with 1.4% not specifying the severity (Table 1). Pain prevalence exhibited significant variations across sociodemographic groups (Table 2). Rural residents reported a notably higher prevalence (61.27%) compared to urban residents (52.19%, *p* < 0.001). A clear age gradient was observed, with pain prevalence increasing from 52.36% in the 60–64 age group to 61.00% in those aged 80 and above (*p* < 0.001). Gender disparities were evident, with females experiencing a significantly higher prevalence (62.36%) than males (50.27%, *p* < 0.001). Education level showed an inverse relationship with pain prevalence. Those with no formal education reported the highest pain prevalence (65.08%), followed by those with primary school education (57.98%), while those with junior high school education or above reported the lowest prevalence (46.42%, *p* < 0.001). Lifestyle factors were also associated with pain prevalence. Interestingly, never-smokers reported a higher prevalence of pain (57.98%) compared to regular smokers (52.62%, *p* < 0.001). Physical activity showed a protective effect, with those who never exercised reporting a higher pain prevalence (61.47%) compared to those who exercised six or more times a week (49.51%, *p* < 0.001).

### 3.3. Geographic Variations in Pain Prevalence

**Figure 2:**
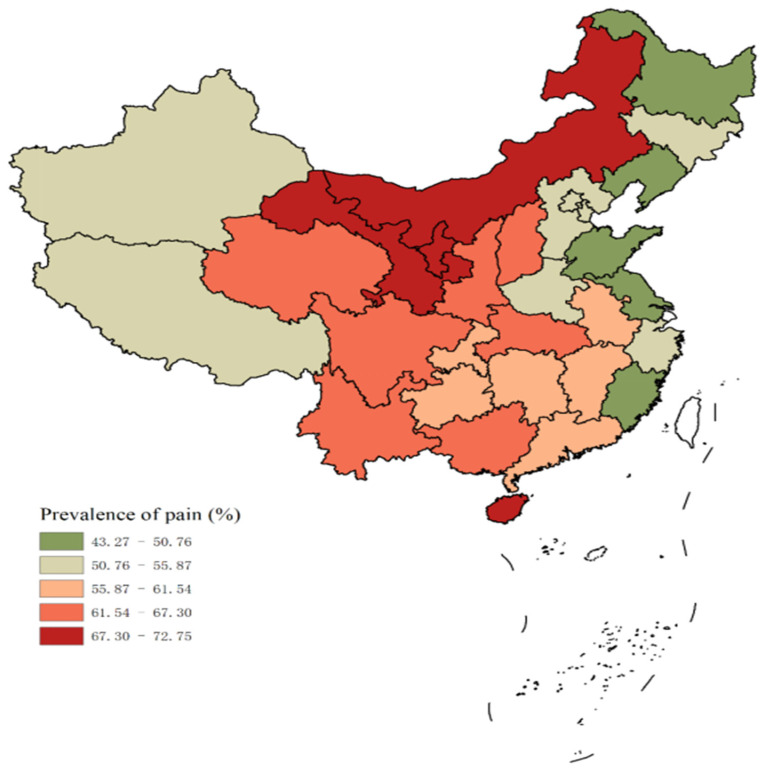
Prevalence of pain at the provincial level.

**Figure 3:**
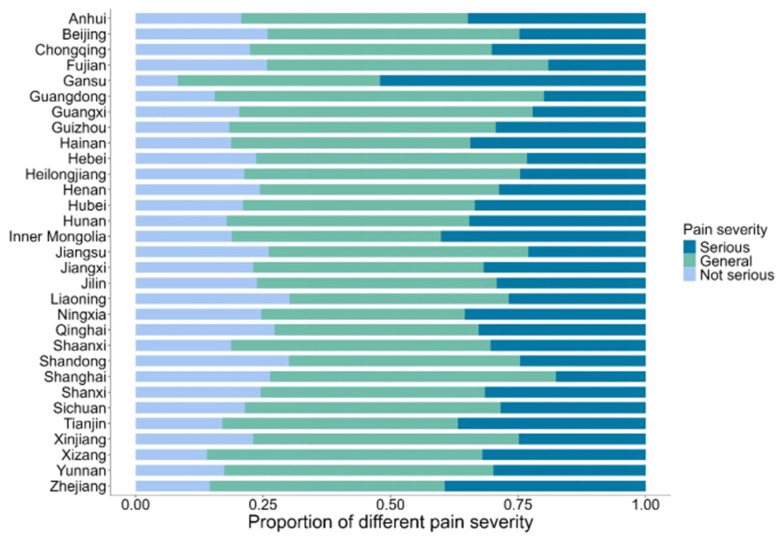
Proportion of pain severity in each province.

Substantial geographic variations in pain prevalence were observed across Provinces in China, as illustrated in Figure 2 & Table A1 (Appendix A). The prevalence ranged from 38.98% (95% *CI*: 34.28–43.69%) in Shanghai to 72.75% (95% *CI*: 67.98–77.53%) in Gansu Province. Generally, western China had a higher pain prevalence than eastern China. The proportion of severe pain also varied geographically (Figure 3). Gansu Province had the highest overall prevalence and the highest proportion of severe pain (37.72%), while Shanghai reported the lowest proportion (6.78%). This pattern suggests that the frequency and intensity of pain experiences differ across regions.

### 3.4. Factors Associated with Pain

**Figure 4:**
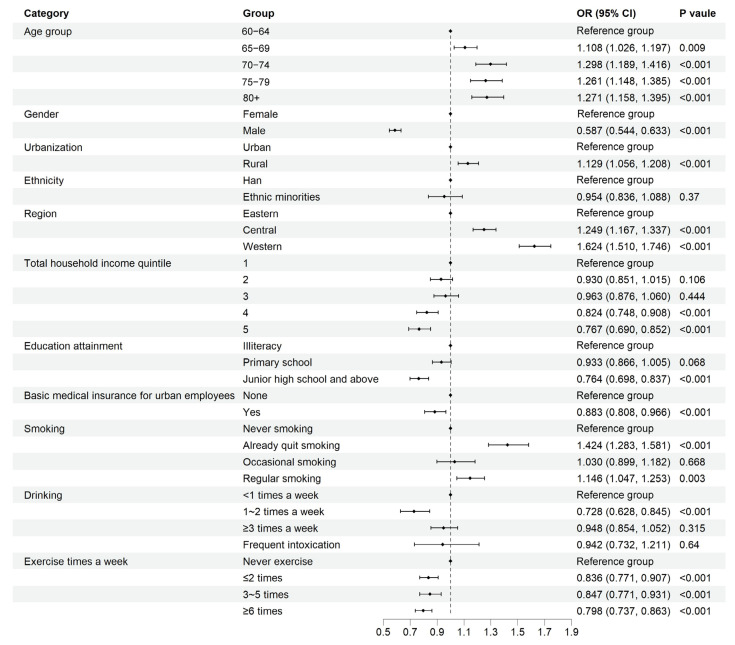
Socioeconomic determinants of pain among Chinese older adults.

Multivariate logistic regression analysis revealed several factors significantly associated with pain (Figure 4). Compared with the 60–64 age group, all older age groups had higher odds of pain. The 70–74 age group demonstrated the highest odds (*OR* = 1.298, 95% *CI*: 1.189–1.416, *p* < 0.001). Gender played a crucial role, with males having substantially lower odds of pain than females (*OR* = 0.587, 95% *CI*: 0.544–0.633, *p* < 0.001). Rural residence was associated with higher odds of pain (*OR* = 1.129, 95% *CI*: 1.056–1.208, *p* < 0.001). Regional differences were pronounced, with both central (*OR* = 1.249, 95% *CI*: 1.167–1.337, *p* < 0.001) and western regions (*OR* = 1.624, 95% *CI*: 1.510–1.746, *p* < 0.001) showing higher odds of pain compared with the eastern region. Education level demonstrated a protective effect, with those having junior high school education and above experiencing lower odds of pain than those with no formal education (*OR* = 0.764, 95% *CI*: 0.698–0.837, *p* < 0.001). Interestingly, former smokers had higher odds of pain than never smokers (*OR* = 1.424, 95% *CI*: 1.283–1.581, *p* < 0.001), while regular exercise was consistently associated with lower odds of pain across all frequency categories.

### 3.5. Chronic Diseases and Pain

**Figure 5:**
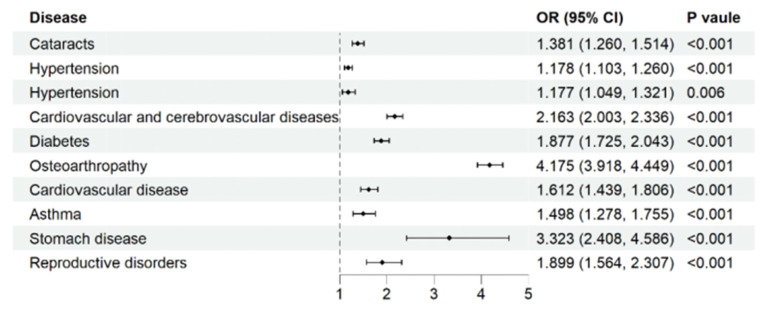
Association between chronic diseases and pain. Note: Adjusted for the following information factors: age group, gender, urbanization, ethnicity, region, total household income quintile, education attainment, basic medical insurance for urban employees, smoking, drinking, exercise times a week.

All investigated chronic diseases were significantly associated with higher odds of pain (Figure 5). Osteoarthropathy showed the strongest association (*OR* = 4.175, 95% *CI*: 3.918–4.449, *p* < 0.001), followed by stomach disease (*OR* = 1.877, 95% *CI*: 1.725–2.043, *p* < 0.001) and cardiovascular and cerebrovascular diseases (*OR* = 2.163, 95% *CI*: 2.003–2.336, *p* < 0.001). Other conditions, such as asthma, diabetes, and cancer, also demonstrated significant associations with pain.

**Figure 6:**
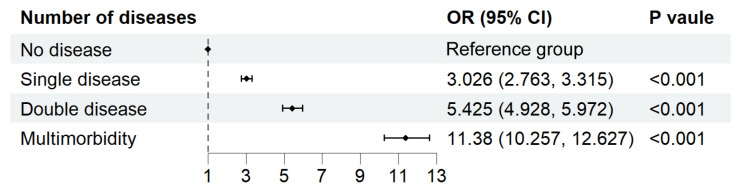
Association between number of chronic diseases and pain. Note: Adjusted for the following basic information factors: age group, gender, urbanization, ethnicity, region, total household income quintile, education attainment, basic medical insurance for urban employees, smoking, drinking, exercise times a week.

A clear and striking trend of increasing pain prevalence was observed with the number of chronic conditions (Figure 6). Compared to those without any chronic disease, the odds of pain were 3.026 times higher (95% *CI*: 2.763–3.315, *p* < 0.001) for those with a single chronic disease. This risk escalated dramatically for those with two chronic diseases (*OR* = 5.425, 95% *CI*: 4.928–5.972, *p* < 0.001) and reached its peak for those with multimorbidity, defined as three or more chronic diseases (*OR* = 11.380, 95% *CI*: 10.257–12.627, *p* < 0.001). This dose–response relationship underscores the cumulative impact of multiple chronic conditions on pain experiences in older adults.

## 4. Discussion

This large-scale, nationally representative study provides comprehensive insights into the prevalence, geographic variations, and determinants of pain among older adults in China. Our findings reveal a high overall prevalence of pain (56.5%) among Chinese older adults, demonstrating the significant burden of this health issue in the world’s most populous nation. This prevalence, however, is not uniformly distributed across the population. Significant sociodemographic disparities emerged, with rural residents, females, and those with lower education levels experiencing disproportionately higher rates of pain. These disparities extend to geographic variations, as we observed substantially higher pain prevalence in western regions compared to eastern areas, highlighting potential inequalities in healthcare access and socioeconomic factors. Furthermore, our analysis uncovered strong associations between chronic diseases and pain, particularly for conditions such as osteoarthropathy, stomach diseases, and cardiovascular ailments. Notably, we identified a clear dose–response relationship between the number of chronic conditions and the likelihood of experiencing pain, underscoring the complex interplay between multimorbidity and pain in older adults.

The overall prevalence of pain in our study aligns with previous estimates from China. A survey in China shows that the prevalence of chronic pain exceeds 60% among elderly individuals aged 60–70, and over 80% among those aged 70 and above [22]. Our results are also consistent with the range reported in international literature, which estimates pain prevalence among older adults between 36.9% and 66% [13,23,24,25]. Our results corroborate previous research demonstrating higher pain prevalence among rural residents, females, and individuals with lower educational attainment [26]. The observed increase in pain prevalence with age aligns with the cumulative effect of age-related health conditions and underscores the need for age-specific pain management strategies.

Our study reveals significant geographic variations in pain prevalence, with higher rates observed in western regions of China. This pattern likely reflects the influence of regional socioeconomic disparities and inequitable distribution of healthcare resources [27,28]. The elevated pain prevalence in rural areas and less economically developed regions suggests potential inadequacies in pain management resources and interventions across different areas of the country [29]. These findings highlight the need for targeted policy measures to improve pain management in underserved areas. Such measures may include enhancing healthcare infrastructure, improving access to pain specialists, and implementing region-specific pain management programs [30]. Future research should aim to elucidate the specific factors contributing to these regional differences, including environmental, cultural, and healthcare system variations.

A notable finding of our study is the robust dose–response relationship between the number of chronic conditions and the odds of experiencing pain. The odds of pain increased dramatically with each additional chronic condition, reaching an odds ratio of 11.380 (95% *CI*: 10.257–12.627) for individuals with three or more chronic diseases compared to those without any chronic conditions. This finding quantifies the cumulative impact of multimorbidity on pain prevalence in a large, diverse sample of older adults, extending previous research in this area [31]. The strong association between multimorbidity and pain underscores the necessity for integrated care approaches that address pain management within the broader context of chronic disease management. Healthcare providers should be cognizant of the elevated likelihood of pain in patients with multimorbidity and incorporate comprehensive pain assessment and management strategies into their care plans. Future research efforts should focus on developing and evaluating multidisciplinary interventions that can effectively manage pain in the context of multiple chronic conditions.

Our analysis also revealed strong associations between specific chronic diseases and pain, with osteoarthropathy, stomach diseases, and cardiovascular conditions showing the most robust links. These findings are consistent with previous studies that have identified these conditions as common sources of pain in older adults [32,33,34]. The particularly strong association with osteoarthropathy emphasizes the importance of musculoskeletal health in pain management for this population.

These findings have direct clinical implications. Healthcare providers should prioritize pain screening for high-risk groups: older women with multimorbidity, rural residents, and those with limited education. Pain assessment should be integrated as the “fifth vital sign” in chronic disease management, particularly for patients with multiple conditions who showed 11-fold higher odds of pain. Our data support prescribing regular exercise as first-line therapy, given its protective effect (*OR* = 0.62 for ≥6 times/week). For the 13 provinces with >60% pain prevalence, targeted interventions including provider training in culturally appropriate assessment tools and community-based exercise programs are urgently needed.

Several limitations of our study warrant consideration. The cross-sectional design precludes causal inferences about the relationships observed. The reliance on self-report using a verbal rating scale rather than standardized instruments may introduce recall bias and subjective interpretation of pain severity. The categorization of pain as mild, moderate, or severe was based on participants’ subjective assessments without standardized pain intensity scales (such as VAS or NRS), which may have resulted in measurement variability across individuals. Additionally, our study did not assess the duration, frequency, or impact of pain on daily activities, which could provide further insights into the burden of pain. Future longitudinal studies are needed to elucidate the causal relationships between chronic diseases, multimorbidity, and pain. Qualitative research exploring the lived experiences of older adults with pain could also provide valuable insights to inform patient-centered interventions.

Our findings have important implications for China’s healthcare system transformation. The strong association between multimorbidity and pain suggests that traditional disease-specific care models may be inadequate for managing pain in older adults. Instead, patient-centered integrated care models that simultaneously address multiple chronic conditions and their associated pain symptoms are needed. The geographic disparities we identified align with China’s broader health equity challenges and suggest that pain management resources should be prioritized in national health poverty alleviation programs. Furthermore, the higher pain burden among women and those with lower education indicates the need for tailored health literacy programs and gender-sensitive clinical protocols. As China continues to develop its primary healthcare system and long-term care insurance schemes, incorporating comprehensive pain assessment and management should be considered a key quality indicator for elderly care services.

## 5. Conclusions

This nationally representative study of 21,346 older adults demonstrates that pain affects 56.5% of Chinese adults aged 60 and above, representing approximately 140 million individuals nationwide. The study reveals significant sociodemographic disparities, with higher prevalence among rural residents (61.27%), females (62.36%), and those with limited education (65.08%). Geographic variations range from 38.98% in Shanghai to 72.75% in Gansu Province, reflecting unequal healthcare resource distribution. Most notably, a strong dose–response relationship exists between multimorbidity and pain, with odds ratios increasing from 3.03 for single chronic disease to 11.38 for three or more conditions.

These findings underscore the urgent need for integrated pain management strategies within China’s healthcare system. Priority interventions should include incorporating pain assessment and treatment protocols into chronic disease management frameworks, particularly for multimorbid patients. Regional disparities necessitate targeted resource allocation to underserved western and rural areas, including enhanced provider training and improved access to specialized pain services. Gender-responsive and education-tailored approaches are essential for addressing the disproportionate burden among vulnerable populations.

In conclusion, pain represents a substantial public health challenge for China’s aging population, with pronounced disparities across demographic and geographic dimensions. Addressing this burden requires multisectoral approaches combining clinical integration, health system strengthening, and equity-focused policies. Future research should employ longitudinal designs to establish causal relationships and evaluate integrated care models, ultimately contributing to evidence-based strategies for promoting healthy aging in China and other rapidly aging societies.

## Figures and Tables

**Table 1:** Basic information of the study population.

Category	Group	Number (%)
Total		21,346 (100)
Pain	None	9280 (43.5)
	Yes	12,066 (56.5)
Pain degree	Mild	2577 (21.4)
	Moderate	5877 (48.7)
	Severe	3438 (28.5)
	Missing	174 (1.4)
Age group	60–64	7277 (34.1)
	65–69	4795 (22.5)
	70–74	3296 (15.4)
	75–79	2850 (13.4)
	≥80	3128 (14.7)
Gender	Female	11,042 (51.7)
	Male	10,304 (48.3)
Urbanization	Urban	11,162 (52.3)
	Rural	10,184 (47.7)
Ethnicity	Han	20,036 (93.9)
	Ethnic minorities	1294 (6.1)
	Missing	16 (0.1)
Region	Eastern	8973 (42.0)
	Central	6529 (30.6)
	Western	5844 (27.4)
Total household income quintile	1	4342 (20.3)
	2	4804 (22.5)
	3	3692 (17.3)
	4	4475 (21.0)
	5	3829 (17.9)
	Missing	204 (1.0)
Education attainment	Illiteracy	6069 (28.4)
	Primary school	8863 (41.5)
	Junior high school and above	6357 (29.8)
	Missing	57 (0.3)
Basic medical insurance for urban employees	None	16,791 (78.7)
	Yes	4498 (21.1)
	Missing	57 (0.3)
Smoking	Never smoking	14,023 (65.7)
	Already quit smoking	2225 (10.4)
	Occasional smoking	1061 (5.0)
	Regular smoking	3938 (18.4)
	Missing	99 (0.5)
Drinking	<1 times a week	18,159 (85.1)
	1~2 times a week	825 (3.9)
	≥3 times a week	1918 (9.0)
	Frequent intoxication	274 (1.3)
	Missing	170 (0.8)
Exercise times a week	Never exercise	10,629 (49.8)
	≤2 times	3482 (16.3)
	3~5 times	2471 (11.6)
	≥6 times	4720 (22.1)
	Missing	44 (0.2)
Cataracts	None	18,006 (84.5)
	Yes	3305 (15.5)
Hypertension	None	13,351 (62.6)
	Yes	7960 (37.4)
Diabetes	None	19,464 (91.3)
	Yes	1847 (8.7)
Cardiovascular disease	None	15,933 (74.8)
	Yes	5378 (25.2)
Stomach disease	None	17,105 (80.3)
	Yes	4206 (19.7)
Arthritis	None	11,318 (53.1)
	Yes	9993 (46.9)
Chronic lung disease	None	19,060 (89.4)
	Yes	2251 (10.6)
Asthma	None	20,150 (94.6)
	Yes	1161 (5.4)
Cancer	None	21,065 (98.8)
	Yes	246 (1.2)
Reproductive disease	None	20,602 (96.7)
	Yes	709 (3.3)
Number of diseases	No disease	3795 (17.8)
	Single disease	6729 (31.5)
	Double diseases	5482 (25.7)
	Multimorbidity	5305 (24.9)
	Missing	35 (0.2)

**Table 2:** Prevalence of pain in each subgroup.

Category	Group	N (%)	χ^2^	*p*
Age group	60–64	3810 (52.36)	105.18	<0.001
	65–69	2672 (55.72)		
	70–74	1973 (59.86)		
	75–79	1703 (59.75)		
	80+	1908 (61.00)		
Gender	Female	6886 (62.36)	317.05	<0.001
	Male	5180 (50.27)		
Urbanization	Urban	5826 (52.19)	178.76	<0.001
	Rural	6240 (61.27)		
Ethnicity	Han	11,278 (56.29)		
	Ethnic minorities	781 (60.36)		
Region	Eastern	4574 (50.98)	235.37	<0.001
	Central	3776 (57.83)		
	Western	3716 (63.59)		
Total household income quintile	1	2759 (63.54)	294.48	<0.001
	2	2886 (60.07)		
	3	2181 (59.07)		
	4	2329 (52.04)		
	5	1806 (47.17)		
Education attainment	Illiteracy	3950 (65.08)	452.76	<0.001
	Primary school	5139 (57.98)		
	Junior high school and above	2951 (46.42)		
Basic medical insurance for urban employees	None	9959 (59.31)	249.94	<0.001
	Yes	2076 (46.15)		
Smoking	Never smoking	8131 (57.98)	55.75	<0.001
	Already quit smoking	1275 (57.3)		
	Occasional smoking	530 (49.95)		
	Regular smoking	2072 (52.62)		
Drinking	<1 times a week	10,478 (57.7)	78.75	<0.001
	1~2 times a week	372 (45.09)		
	≥3 times a week	979 (51.04)		
	Frequent intoxication	146 (53.28)		
Exercise times a week	Never exercise	6534 (61.47)	225.07	<0.001
	≤2 times	1867 (53.62)		
	3~5 times	1308 (52.93)		
	≥6 times	2337 (49.51)		
Cataracts	None	9783 (54.33)	236.19	<0.001
	Yes	2272 (68.74)		
Hypertension	None	7192 (53.87)	105.93	<0.001
	Yes	4863 (61.09)		
Diabetes	None	10,932 (56.17)	14.72	<0.001
	Yes	1123 (60.80)		
Cardiovascular disease	None	8247 (51.76)	593.69	<0.001
	Yes	3808 (70.81)		
Stomach disease	None	8955 (52.35)	626.57	<0.001
	Yes	3100 (73.70)		
Arthritis	None	4451 (39.33)	2919.38	<0.001
	Yes	7604 (76.09)		
Chronic lung disease	None	10,413 (54.63)	274.88	<0.001
	Yes	1642 (72.95)		
Asthma	None	11,173 (55.45)	188.13	<0.001
	Yes	882 (75.97)		
Cancer	None	11,869 (56.34)	36.75	<0.001
	Yes	186 (75.61)		
Reproductive disease	None	11,529 (55.96)	92.70	<0.001
	Yes	526 (74.19)		
Number of diseases	No disease	927 (24.43)	2948.04	<0.001
	Single disease	3389 (50.36)		
	Double diseases	3538 (64.54)		
	Multimorbidity	4201 (79.19)		

All percentages calculated based on non-missing values; *p*-values derived from chi-square tests for categorical variables and Cochran–Armitage trend tests for ordinal variables; Statistical significance: *p* < 0.001. Note: Missing data excluded from percentage calculations but reported in Table 1.

## Data Availability

The data that support the findings of this study are not publicly available due to data ownership and access restrictions. The datasets used in this study are proprietary to the National Center on Gerontology of China and were accessed under a data use agreement that allows for analysis but prohibits public sharing. The authors have permission to use these data for research purposes only and do not have authorization to distribute or share the raw data with third parties. Researchers interested in accessing these data should contact the National Center on Gerontology of China directly for information on data access procedures and requirements. Aggregated results and summary statistics from this study are available from the corresponding author upon reasonable request, subject to the data use agreement limitations.

## Appendix A

**Table A1:** Number of patients and prevalence of pain in each province.

Province	Number of People with Pain	Prevalence of Pain (%)
Anhui	616	57.46 (54.50, 60.42)
Beijing	168	50.76 (45.37, 56.14)
Fujian	243	47.28 (42.96, 51.59)
Gansu	243	72.75 (67.98, 77.53)
Guangdong	746	57.08 (54.39, 59.76)
Guangxi	515	64.21 (60.90, 67.53)
Guizhou	317	58.38 (54.23, 62.53)
Hainan	97	72.39 (64.82, 79.96)
Hebei	534	52.35 (49.29, 55.42)
Henan	766	55.87 (53.24, 58.50)
Heilongjiang	251	46.48 (42.27, 50.69)
Hubei	588	62.96 (59.86, 66.05)
Hunan	682	60.19 (57.34, 63.04)
Jilin	194	52.86 (47.75, 57.97)
Jiangsu	750	49.37 (46.86, 51.89)
Jiangxi	355	57.82 (53.91, 61.72)
Liaoning	363	43.27 (39.91, 46.62)
Inner Mongolia	214	67.30 (62.14, 72.45)
Ningxia	66	72.53 (63.36, 81.70)
Qinghai	56	61.54 (51.54, 71.53)
Shandong	882	50.49 (48.14, 52.83)
Shanxi	324	65.06 (60.87, 69.25)
Shaanxi	340	62.73 (58.66, 66.80)
Shanghai	161	38.98 (34.28, 43.69)
Sichuan	1000	65.10 (62.72, 67.49)
Tianjin	106	55.50 (48.45, 62.55)
Xizang	50	53.76 (43.63, 63.90)
Xinjiang	118	53.39 (46.82, 59.97)
Yunnan	428	64.75 (61.11, 68.39)
Zhejiang	524	54.70 (51.55, 57.85)
Chongqing	369	60.29 (56.42, 64.17)
